# Society Meetings

**Published:** 1906-02

**Authors:** 


### SOCIETY MEETINGS.

The Buffalo Academy of Medicine held meetings during the month of January as follows:

*Section of Surgery.*—Tuesday, January 2, 1906, at 8:30 P. M. Program: What do recent researches concerning the ductless glands teach regarding the treatment of exophthalmic goitre, Roswell Park.

*Section of Medicine.*—Tuesday, January 9, 1906, at 8:30 P. M. Program: (a) The relation of alcoholism to tuberculosis, Joseph W. Grovesnor; discussion opened by Dr. DeLancey Rochester. (a) Two unusual cases of nephritis, George A. Himmelsbach; discussion opened by Thomas B. Carpenter.

*Section of Obstetrics and Gynyecology.*—Tuesday, January 23, 1906, at 8:30 P. M. Program: The pernicious vomiting of pregnancy, Mathew D. Mann. Pubiotomy, Max Breuer.

A stated meeting of the Academy was held Tuesday, January 16, 1906, at 8:30 P. M. The program was furnished by the

Pathological Section, as follows: Bacteriolysis of hog's blood, B. Meade Bolton, Washington D. C. A collation was served at the close of the meeting.

---

THE Alumni Association of the Medical Department of the University of Buffalo, through its executive committee, is making extraordinary efforts to ensure an unusually attractive meeting during the forthcoming commencement week, which begins May 28, 1906.

---

THE American Association of Obstetricians and Gynecologists will hold its Nineteenth Annual Meeting at Cincinnati, Thursday, Friday and Saturday, September 20, 21 and 22, 1906, under the administration of Dr. John Young Brown, president, of Saint Louis.

